# Pulmonary Vascular Proliferative Lesions in Wild Korean Raccoon Dogs (*Nyctereutes procyonoides*): Description of 13 Cases

**DOI:** 10.3390/vetsci13010021

**Published:** 2025-12-24

**Authors:** Warisraporn Tangchang, Jun-Yeop Song, Do-hyun Kim, Hyo-Jung Kwon, Hwa-Young Son

**Affiliations:** College of Veterinary Medicine, Chungnam National University, Daejeon 34134, Republic of Korea; waris0770@gmail.com (W.T.);

**Keywords:** intravascular papillary proliferative lesion, immunohistochemistry, scabies, heartworm, pathology, Korean raccoon dogs

## Abstract

Pulmonary vascular proliferative lesions are rarely reported and poorly characterized in animals. In this study, we describe 13 cases identified in wild Korean raccoon dogs (*Nyctereutes procyonoides*), suggesting a higher-than-expected incidence in this species. Gross examination revealed villous projections within the lumina of pulmonary vessels, sometimes accompanied by pneumonia, hemorrhage, or *Dirofilaria immitis* (heartworm) infection. Most affected animals also presented with thick, dark gray cutaneous crusts associated with scabies infestation. Histopathologically, the lesions consisted of papillary proliferations within thickened vascular lumens. Special stains (Masson’s trichrome and Elastic Verhoeff–Van Gieson) demonstrated a single layer of endothelial cells lining fibromuscular and collagenous thick cores. Immunohistochemistry confirmed endothelial origin and benign proliferative nature, with positive expression of CD31, collagen types I, III, and IV, and proliferating cell nuclear antigen (PCNA). To date, pulmonary vascular proliferative lesions have not been well documented in *N. procyonoides*, and baseline pathological data, including findings from special stains, are lacking. These findings indicate that pulmonary vascular proliferative lesions may be underrecognized in raccoon dogs and suggest a likely association with chronic vascular injury related to parasitic infections. Further studies are warranted to elucidate the underlying mechanisms and contributing factors.

## Simple Summary

Pulmonary vascular growths inside blood vessels are unusual in animals, and very little is known about them. In this study, we examined 13 wild Korean raccoon dogs that were found to have these abnormal tissue growths in the lungs. We aimed to understand what these growths look like, what may cause them, and whether they are connected to other health problems. We found that many raccoon dogs had small finger-like projections growing inside the lung blood vessels. Most of these animals were also infected with parasites, such as heartworms in the chest and mites on the skin, which can cause long-term irritation and vasculitis, respectively. Under the microscope, the growths appeared to be made of harmless (non-cancerous) cells forming small papillary structures. These findings suggest that long-term irritation from parasitic infections may contribute to these unusual lung lesions. This information is valuable for wildlife health monitoring and helps veterinarians and researchers better understand diseases affecting raccoon dogs.

## 1. Introduction

Intravascular papillary endothelial proliferation is a rare and not fully specified disease with limited reported cases in animals. We have previously reported on the lesions of the pulmonary vessels of wild Korean raccoon dogs [1]. In that study, scabies with hair loss was observed on the raccoon’s body, but no heartworm infections were noted. Grossly, the lung was larger than normal size, contained white villous projections in the vascular lumen, and was histopathologically characterized by luminal dilation, thickening, and villous papillary hyperplasia of the pulmonary vessels [1]; however, the pathophysiology of this lesion remained ambiguous. Therefore, we collected additional raccoon cases for further pathological analysis to better understand the pathogenesis and characteristics of pulmonary lesions in wild Korean raccoon dogs. The illustration of vascular initiation and proliferative index through immunohistochemical (IHC) could encourage precise differential diagnosis [2]. Vascular initiation and the identification of IHC markers in the three layers of blood vessels—tunica intima (CD31 (platelet endothelial cell adhesion molecule-1 [PECAM-1]) and von Willebrand factor (vWF) [3,4], collagen 4 [5]), tunica media (alpha-smooth muscle actin [α-SMA], vimentin [5]), and tunica externa or adventitia (collagen 1, 3, and 4) [5]—play a crucial role in understanding the pathology of vascular lesions such as IPEH. By targeting these specific IHC markers, it becomes possible to map the cellular composition and pathological changes across the three layers of blood vessels, aiding in the diagnosis and understanding of vascular lesions like IPEH. The use of special stains, such as Masson’s trichrome and Elastic Verhoeff’s Van Gieson (EVG), further enhances the visualization of collagen and elastin fibers, contributing to a comprehensive analysis of vascular abnormalities. The aim of this study was to demonstrate additional diagnostic features of IPEH using special stains and enhance understanding of the pathogenesis of pulmonary lesions in raccoons.

## 2. Materials and Methods

### 2.1. Post-Mortem Examination and Sample Collection

In 2022–2023, complete post-mortem examinations were conducted on thirteen raccoon dog carcasses at the Daejeon Wildlife Rescue Center. Multiple sections of lung tissue and abnormal samples from the heart, liver, kidneys, and other organs were collected, examined, and recorded.

### 2.2. Histological and Immunohistochemical Analysis

All lung and abnormal tissues were fixed in 10% neutral buffered formalin, processed through routine paraffin embedding, and sectioned at 4 µm thickness. For histopathological analysis, the paraffin-embedded sections were stained with hematoxylin and eosin (H&E) for routine evaluation. Additional specialized staining, including Masson’s trichrome, EVG, and IHC, was performed on lung tissue samples to assess the lesions in detail.

For IHC, detailed antibody information is provided in Table 1. Deparaffinized sections were processed using the Avidin–Biotin Complex (ABC) method (The VECTASTAIN^®^ Elite^®^ ABC Universal Kit (Peroxidase; Vector Laboratories, Newark, CA, USA)). High-temperature antigen retrieval was performed using citrate buffer (pH 6.0) to unmask antigenic sites. Endogenous peroxidase activity was quenched by incubating the sections in 0.3% hydrogen peroxide in distilled water for 15 min.

Sections were blocked with diluted normal serum for 120 min in a humidified chamber and then incubated overnight at 4 °C with primary antibodies diluted in normal serum. The following day, slides were washed with phosphate-buffered saline (PBS) and incubated with an appropriate biotinylated secondary antibody at 37 °C for 120 min. Next, the sections were incubated with the ABC reagent for another 30 min.

The antibody–antigen binding was visualized using 3,3′-diaminobenzidine (DAB) as a chromogen (DAB Peroxidase Substrate Kit (Vector Laboratories, Newark, CA, USA)), resulting in brown-colored staining, and counterstained with hematoxylin to highlight cellular architecture.

Negative controls were prepared by omitting the primary antibody, while normal vascular cell components served as internal positive controls to ensure staining validity. Pathological features were assessed comprehensively, evaluating staining intensity and specific cellular localization to ensure a thorough histological and immunohistochemical interpretation.

### 2.3. Immunoreactivity Scoring and Interpretation

Immunoreactivity markers were evaluated semi-quantitatively based on the intensity of staining observed under the microscope. The staining intensity was graded using the following scale: – = Negative (no staining observed); + = Mild (weak or faint staining); ++ = Moderate (clear and intermediate staining intensity); and +++ = Strong (intense staining throughout the target area).

## 3. Results

### 3.1. Post-Mortem and Routine Histological Lesions

**Figure 1:**
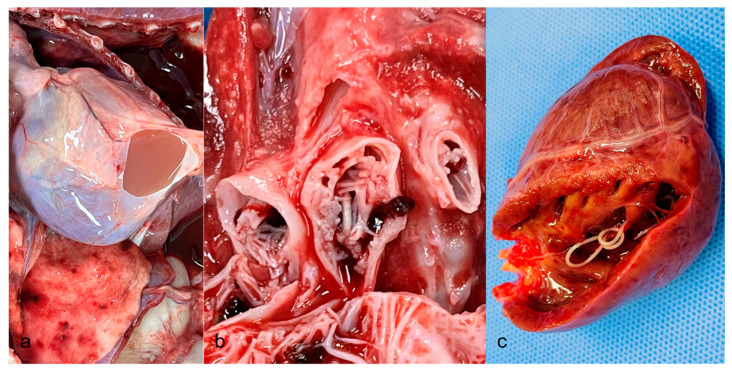
Gross findings in the raccoons with cardiopulmonary involvement. (**a**) Hydropericardium and multifocal dark-red discolored spots in the lung. (**b**) Villous hyperplasia in the lumen and dilated pulmonary vessels. (**c**) A heartworm is found in the ventral chamber.

**Figure 2:**
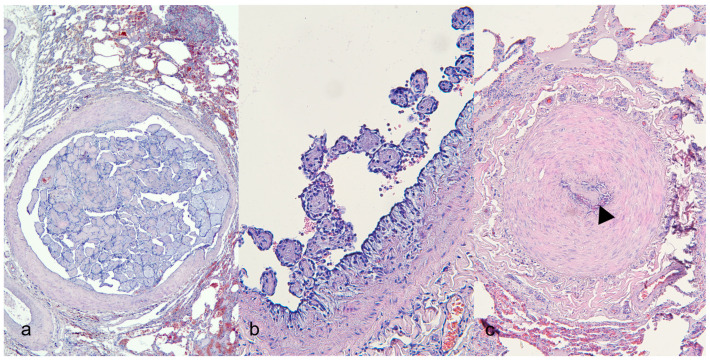
Histopathologic characteristics of pulmonary vessels in raccoons with intravascular papillary endothelial proliferation. Hematoxylin and eosin. (**a**) Dilated vessels with abundant papillary structures within the vascular lumen; hyperplasia of the tunica media layer is observed (4×). (**b**) The basement membrane of the endothelial layer is swollen, and some papillary structures consist of matrix components derived from the intimal area (20×). (**c**) The pulmonary artery shows papillary proliferations (arrowhead) packed into the intravascular space, along with extensive muscular hypertrophy (10×).

The twelve carcasses exhibited diffuse alopecia with severely thickened, hyperpigmented skin and moist crusts, while one carcass showed normal skin without crusts. Necropsy findings revealed prominent pulmonary lesions, including pneumonia, hemorrhage, and villi-like projections on the inner surface of the pulmonary vessels (Figure 1a). Additionally, accumulation of serous fluid in the pericardium (Figure 1b) and the presence of heartworms in the right ventricular chambers (Figure 1c) were noted in some cases. No significant pathological changes were detected in other organs. Interestingly, one raccoon exhibited good body condition with normal skin and healthy lungs; however, heartworms were present in the right ventricular chambers (Table 2). Microscopic examination of the skin revealed severe hyperkeratosis and numerous burrows containing mites within the epidermis. In the lungs, unique pathological features were identified, including proliferative lesions (Figure 2a), fibromuscular proliferation (Figure 2b), medial hypertrophy and hyperplasia, and muscular hypertrophy (Figure 2c). Routine H&E staining confirmed these findings. Nonspecific lesions, such as inflammatory cell infiltration and pigmentation, were observed in other organs, including the liver, kidneys, heart, and brain. A summary of the observed pulmonary vascular lesions is presented in Table 3. Proliferation was consistently identified in large vessels located near the bronchi or bronchioles, as well as in small to medium-sized vessels. Muscular hypertrophy was predominantly noted in cases with infection, and importantly, no thrombosis was observed in any of the examined specimens.

### 3.2. Special Stains and Immunohistochemical Characterization of Proliferative Lesions

**Figure 3:**
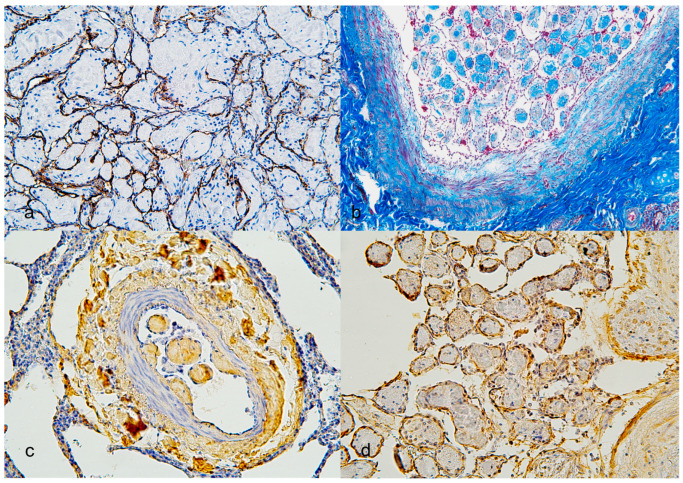
Papillary features in the vascular space. (**a**) Cytoplasmic expression of anti-CD31 in a single layer of endothelial cells. (20×; Immunohistochemistry, IHC). (**b**) The papillary structures and vascular layer show abundant blue collagen accumulation. (10×; Masson’s trichrome). (**c**) The tunica adventitia area and small papillary protrusions strongly react to the collagen 3 antibody (20×; IHC). (**d**) Collagen 4 is prominently observed beneath the basement membrane of endothelial cells. (20×; IHC).

**Figure 4:**
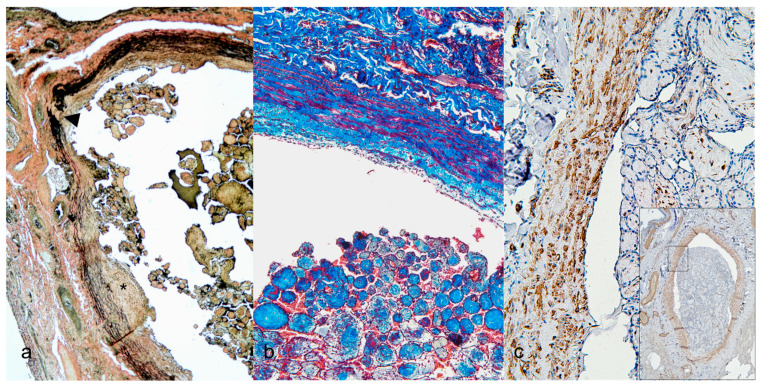
Vascular layer manners of pulmonary intravascular papillary endothelial proliferation. (**a**) Some ridges show intimal thickening and hyperplasia of lining endothelial cells and increase the distance between intima and internal elastic lamina (asterisk), disruption of the internal elastic lamina and elastic fibers (arrowhead) (10×; Elastic Verhoeff’s Van Gieson). (**b**) The papillary part and vascular layer show numerous red muscular and fibromuscular cytoplasm (10×; Masson trichrome). (**c**) Scattered muscular and fibromuscular cells are positive for alpha-smooth muscle actin in the vascular layer and papillary areas (20×; immunohistochemistry); inset image, 4×.

Special stains, including Masson’s trichrome, EVG, and IHC, were performed to further characterize the lesions described in Table 3. Based on the staining results of Endothelial Cell Coverage, all proliferative areas were confirmed to be covered by a single layer of endothelial cells, as evidenced by strong CD31(Figure 3a) and vWF immunoreactivity. For Collagen Identification, the collagen composition of the lesions was confirmed through Masson’s trichrome staining, which highlighted the presence of abundant, blue-stained collagen within the lesion (Figure 3b). IHC for collagen types 1 and 3 showed strong positivity within the lesion (Figure 3c), while collagen type 4 was prominently expressed beneath the endothelial layer, correlating with the basement membrane zone (Figure 3d). Elastic Lamina and Intimal Changes. EVG staining revealed an increased distance between the intima and the internal elastic lamina, evidenced by disrupted or stretched black elastic fibers (Figure 4a). CD31 and vWF confirmed endothelial proliferation within this altered region. For Fibromuscular Proliferation, an increased number of muscle and fibromuscular cells and their associated areas were identified using Masson’s trichrome staining, which highlighted red-stained muscular components in the papillary projections (Figure 4b). IHC for α-SMA (Figure 4c) and vimentin, which demonstrated significant fibromuscular proliferation within the intimal layer. Proliferative Activity was observed to assess the proliferative nature of the lesion and proliferating cell nuclear antigen (PCNA) immunostaining was performed. The results showed low expression levels, indicating that this pulmonary lesion was benign and not highly proliferative (Table 4).

## 4. Discussion

Proliferative lesions have been reported in various animals, including horses [6], dogs [7,8], and cats [9]. In these cases, intravascular papillary endothelial hyperplasia (IPEH) was primarily observed in non-pulmonary vessel organs, such as the conjunctiva, intramuscular tissues, or subcutaneous masses. In our previous study, pulmonary vascular lesions in wild raccoon dogs were diagnosed as intravascular papillary endothelial hyperplasia (IPEH), while other cases were associated with heartworm infection. Re-evaluation in the present study indicates that these lesions are more appropriately classified as papillary intimal proliferation, based on the presence of papillary intimal projections and the absence of an organized intraluminal thrombus, a defining feature of IPEH. These findings suggest a reactive intimal process rather than true IPEH. This diagnosis was based on a comparative analysis of the histopathological features of the proliferation previously described in humans and other animal species. The findings in raccoons demonstrated similar characteristics, including luminal proliferation of endothelial cells forming papillary structures within the affected blood vessels. We investigated nematode infections in animals and identified similarities between these lesions and IPEH. Such lesions have been referred to as villus intimal proliferation (VIP), intimal proliferation, or villus proliferation in species including dogs [10,11], cats [12,13], and raccoons [11,14]. Additionally, VIP has been reported in capybaras (*Hydrochoerus hydrochaeris*) infected with *Cruorifilaria fuberocauda*, a parasite known to cause significant pulmonary vascular damage [15]. IPEH is characterized by papillary proliferations filling most of the intravascular space; papillary structures comprising collagenous cores lined by a single layer of endothelial cells; presence of thrombi; and absence of significant pleomorphism or notable mitotic activity, which distinguishes IPEH from malignant vascular lesions [6,7,8]. Terms like VIP and villus proliferation often describe thickening and villus-like proliferations of collagen fibers specifically within the intimal layer of the pulmonary vessels [11,12]. To improve the consistency and precision of terminology, we propose the use of “VIP” in this study to describe these lesions. The term “Papillary intimal proliferation” appropriately encompasses cases associated with *Dirofilaria immitis* (DI) infection in animals, vascular malformations [6], traumatic vascular injury [8], and vascular tumor-like lesions reported in various organs [7].

Additionally, based on the true papillary structures observed in our raccoon dog case, which share similar characteristics to IPEH in humans and previously reported cases in animals where DI was not implicated, we believe the term Papillary intimal proliferation is appropriate. The pathogenesis of IPEH remains poorly defined and not fully understood [16,17]. To address this, we analyzed thirteen raccoon dog cases to clarify the underlying mechanisms and estimate the incidence of the proliferative lesion. Our analysis focused on two potential contributors: pulmonary inflammation and vascular injury. DI infection has previously been suggested as a pathophysiological mechanism for proliferative vascular lesions in the lungs. However, both our previous report [1] and the current study demonstrated that heartworms were absent in all carcasses upon both gross and microscopic examination. These findings suggest that heartworm infection alone may not be the sole causative factor of the proliferations. Instead, we hypothesize that the increasing occurrence of it in raccoon dogs may be influenced by septicemia [18] or secondary bacterial infections [18,19,20] related to mangy skin caused by *Sarcoptes scabiei*. The consistent gross findings of lymphadenopathy in all scabies-affected raccoon dogs further support the presence of concurrent or underlying infectious processes. There is pathological evidence indicating a correlation between inflammation and vascular wall proliferation or hypertrophy [21]. In one previous study on raccoon dogs with scabies and local or systemic inflammation, histological examination of the lungs revealed pneumonia and, in some cases, alveolar or bronchial smooth muscle hypertrophy [19]. While that study suggested a potential association between scabies and lung abnormalities [19], our previous report described raccoon dogs with both scabies and IPEH [1]. However, in the current investigation, we have not established a definitive association between scabies and IPEH. To investigate the pathological features of the proliferative lesion, specific staining methods were utilized to examine its diagnostic characteristics [22]. Our findings confirmed that five cases of the lesion were benign proliferative lesions consisting of a single layer of endothelial cells lining the papillary structures. A proliferative fibrous matrix positive for fibromuscular cells and collagen types 1, 3, and 4. These results were based on careful histological examination complemented by IHC. Blood vessel walls are histologically organized into three distinct layers, each with a unique cellular composition and connective tissue components that contribute to their structural and functional integrity [5,23]. Tunica intima (Inner layer) is the innermost layer of the blood vessel wall, lined by a single layer of endothelial cells. It is separated from the tunica media by the internal elastic lamina, which primarily contains collagen type 4 [5]. The collagen type 4 in the basement membrane plays a critical role in supporting endothelial cells and maintaining the structural integrity of the intima. Tunica media (Middle layer) is composed of smooth muscle cells, elastic fibers, and extracellular matrix components. This layer lies beneath the internal elastic lamina and provides the vessel with its contractile and elastic properties. Smooth muscle cells play a central role in maintaining vascular tone and responding to injury through hypertrophy or hyperplasia. Tunica adventitia (Outer layer) extends beyond the external elastic lamina and consists predominantly of collagen types 1 and 3, along with fibroblasts and various connective tissue proteins [5]. In this study, the papillary structures and villous projections within the lesions demonstrated blue collagen expression using Masson’s trichrome stain. IHC confirmed the presence of α-SMA, vimentin, and collagen types 1, 3, and 4, all of which were covered by a single layer of endothelial cells showing strong positive immunostaining for CD31 and vWF. These findings align with previous studies, which also reported the expression of CD31, vimentin, α-SMA, and collagen type 4 within the papillary regions of the lesion [1,22,24]. In our study, the strong CD31 and vWF staining confirmed that the papillary projections consisted of one endothelial cell layer, further supporting the benign nature of the lesion. Additionally, PCNA immunostaining, used to evaluate cellular proliferation, revealed low expression levels. The proliferative index was less than 1%, indicating that the lesions exhibited minimal endothelial proliferation, further classifying the lesion as a benign vascular lesion with limited reactive potential. Further analysis of collagen type 4 demonstrated strong expression beneath the basement membrane in both normal blood vessels and papillary lesions, correlating with its known localization within the intimal layer of the vessel wall [4]. The EVG staining revealed areas of disruption within the internal elastic lamina and increased distance between the intima and the elastic fibers, suggesting structural remodeling of the vessel wall. The immunoreactivity of α-SMA and vimentin highlighted the presence of fibromuscular cells within the papillary structures and villous projections. The α-SMA staining supported the observation of muscular hypertrophy, medial hypertrophy, and hyperplasia, which were routinely identified on H&E-stained sections. The combined findings, including the expression of collagen type 4 and the results from Masson’s trichrome staining, provide robust evidence that the observed lesion represents intimal proliferation. These observations enhance the diagnostic characterization of this lesion and distinguish it from other vascular proliferative conditions. Based on this study, we conclude that various infections leading to vascular injury could partially explain the pathophysiology of papillary intimal proliferation. Another potential mechanism involves non-malignant endothelial cell proliferation and numerous papillae formations within the vascular lumen, possibly triggered by degeneration and necrosis in a hemorrhagic area [16,17]. Additionally, this lesion may be associated with chronic inflammation or vascular irritation [25]. The exact pathogenesis of IPEH remains unclear; studies in human cases commonly suggest two primary mechanisms: reactive endothelial proliferation or thrombosis [26]. In this study, heartworm infection was present in the raccoon dog cases, and no thrombi were observed in the raccoon dog cases; thus, the term papillary intimal proliferation is considered more appropriate. The findings of this study enhance our understanding of pulmonary proliferative lesions in raccoon dogs but highlight the need for further research to identify the specific causes and pathophysiology of this lesion. Future studies, including larger sample sizes and controlled experiments, are necessary to elucidate the precise etiology and its relationship to other vascular diseases, particularly in wildlife species.

## Figures and Tables

**Table 1:** Antibodies used in immunohistochemistry staining.

Antibody	Type ^1^	Clonality ^2^	Clone or ID	Species	Source of Antibody	Dilution	Expression Area
CD31	E	pAb	NB100-2284	rabbit	Novus Biologicals	1:100	Cytoplasm
vWF	E	pAb	ab6994	rabbit	abcam	1:200	
α-SMA	F	mAb	NBP2-33006	mouse	Novus Biologicals	1:500	
Vimentin	F	mAb	D21H3	rabbit	Cell Signaling Technology	1:100	
Collagen type 1	C	pAb	NBP1-77457	rabbit	Novus Biologicals	1:200	
Collagen type 3	C	mAb	NBP1-05119	mouse	Novus Biologicals	1:100	
Collagen type 4	C	pAb	ab6586	rabbit	abcam	1:500	
PCNA	CP	mAb	D3H8P	goat	Cell Signaling Technology	1:1000	Nucleus

vWF, Von Willebrand Factor; α-SMA, Alpha smooth muscle actin; PCNA, Proliferating cell nuclear antigen. ^1^ Type of markers: E, Endothelial cell; F, Fibroblast cell; C, Collagen; and CP, Cell proliferation. ^2^ mAb, monoclonal antibody, and pAb, polyclonal antibody.

**Table 2:** Pathological findings.

Year	2022						2023						
Animal ID	1	2	3	4	5	6	7	8	9	10	11	12	13
Significant lesion													
Heart													
Cardiomegaly	-	-	-	P	-	-	-	-	-	-	-	-	-
Detected heart worm(s)	-	-	-	-	-	-	-	-	-	-	P	P	P
Dilatation of the posterior vena cava	-	P	-	-	-	-	-	-	-	-	-	-	-
Endocarditis	P	-	-	-	-	-	-	-	-	-	-	-	P
Pericardial fluid	P	-	-	P	P	-	-	-	-	-	-	-	-
Right ventricle enlargement	-	P	-	-	-	-	-	-	-	-	-	-	-
Lung													
Villus like protrusion in vessel lumens	P	-	-	-	-	-	-	-	-	-	-	-	-
Pulmonary edema	-	P	-	-	-	-	-	-	-	-	-	-	-
Pneumonia	-	-	P	P	P	P	P	-	P	P	-	P	-
Diffuse hemorrhage	-	-	-	P	-	-	-	-	-	-	-	-	-
Foamy fluid along trachea	-	-	-	P	-	P	-	-	-	-	-	-	-
Hydrostatic congestion	-	-	-	-	-	-	P	-	P	-	P	-	-
Detected heart worm(s)	-	P	-	-	-	-	-	-	-	-	P	-	P
Petechial hemorrhage	-	-	-	-	-	-	-	P	-	-	-	-	P
Skin													
Scabies infection	P	P	P	P	P	P	P	P	P	P	P	-	P

P is present; - means absent.

**Table 3:** Histological findings in emphasized pulmonary vascular lesions of wild raccoon dogs.

Year	2022						2023						
Animal ID	1	2	3	4	5	6	7	8	9	10	11	12	13
Significant lesion													
Heart	P	P	P	P	P	-	-	-	-	-	P	P	P
Skin (Scabies infection)	P	P	P	P	P	P	P	P	P	P	P	-	P
Lung	P	P	P	P	P	P	P	P	P	P	P	P	P
Muscular hypertrophy	P	-	-	-	-	-	-	-	-	-	-	-	-
Eosinophils and neutrophils infiltration in the intima	-	P	-	-	-	-	-	-	-	-	P	P	-
Endarteritis with infiltration of eosinophils	-	-	-	-	-	-	-	-	-	-	-	P	-
Intravascular nematodes	-	P	-	-	-	-	-	-	-	-	-	-	-
Increased the distance between intima and internal elastic lamina	P	P	-	-	-	-	-	-	-	-	P	P	P
Disruption of the internal elastic lamina	P	P	-	-	-	-	-	-	-	-	P	P	P
Intravascular papillary projection (small and medium-sized vessels)	P	P	-	-	-	-	-	-	-	-	P	-	P
Intravascular papillary projection (large vessel)	P	P	-	-	-	-	-	-	-	-	P	P	P

P is present; - means absent.

**Table 4:** Immunohistochemical staining profiles in pulmonary vascular lesions.

Location	CD31	vWF	α-SMA	Vimetin	PCNA	Cl1	Cl3	Cl4
Endothelial cell layer	++	+	-	+	≤1%	+	-	-
Tunica intima	-	-	-	-	≤1%	+	-	++
Tunica media	-	-	+	+	≤1%	+	+	-
Smooth muscle layer	-	-	++	+	≤1%	-	-	-
Tunica adventitia	-	-	-	-	≤1%	-	++	-
Papillary proliferation	++	+	+	+	≤1%	+	+	+

vWF, Von Willebrand Factor; α-SMA, Alpha smooth muscle actin; PCNA, Proliferating cell nuclear antigen; Cl1, Collagen type 1; Cl3, Collagen type 3; Cl4, Collagen type 4. -, negative; +, mild; and ++, moderate.

## Data Availability

The original contributions presented in this study are included in the article. Further inquiries can be directed to the corresponding author.

## Abbreviations

The following abbreviations are used in this manuscript:

α-SMA	Alpha-Smooth Muscle Actin
DI	*Dirofilaria immitis*
EVG	Elastic Verhoeff’s Van Gieson
H&E	Hematoxylin and Eosin
IHC	Immunohistochemistry
IPEH	Intravascular Papillary Endothelial Hyperplasia
PCNA	Proliferating Cell Nuclear Antigen
VIP	Villus Intimal Proliferation
vWF	von Willebrand factor
